# Erosive pustular dermatosis of the scalp with resolution after initiation of dialysis

**DOI:** 10.1016/j.jdcr.2024.08.029

**Published:** 2024-09-12

**Authors:** Marcus Rossi, Joshua Kentosh

**Affiliations:** Department of Dermatology, University of Illinois College of Medicine Peoria, Peoria, Illinois

**Keywords:** dialysis treatment and resolution for dermatologic conditions, elderly population dermatology, erosive pustular dermatosis of the scalp (EPDS), inflammatory scalp condition

## Introduction

Erosive pustular dermatosis of the scalp (EPDS) is an infrequently encountered inflammatory condition predominantly affecting the elderly population.^1^ Characterized by persistent erosions, crusting, and pustules leading to scarring alopecia, EPDS can present a diagnostic challenge due to its resemblance to a plethora of dermatological diseases such as infections, bullous disorders, and neoplasias.^1^ Pye et al first delineated this condition in 1979, bringing attention to its association with local trauma and the consequent diagnostic and therapeutic complexities it presents.^1^ The etiology of EPDS is often linked to mechanical, physical, or pharmacological insults to the scalp, including surgery, cryotherapy, radiotherapy, and even herpes zoster infections.^1^^,^^2^ Despite its nonspecific histological findings, which evolve from spongiotic pustules to chronic mixed inflammation, EPDS necessitates a high index of suspicion and careful differentiation from other mimicking conditions, such as squamous cell carcinoma and psoriasis.^1^

Conventionally, high-potency corticosteroids have been the mainstay of treatment for EPDS, but their use is marred by frequent recurrences and an increased risk of skin atrophy, particularly challenging in the elderly demographic.^2^ We present a case that may shed light on the etiology and potential therapeutic mechanisms, documenting the unexpected resolution of EPDS following the initiation of dialysis treatment in a patient. This unique finding invites further exploration into the pathophysiological underpinnings of this elusive condition.

## Case report

**Fig 1:**
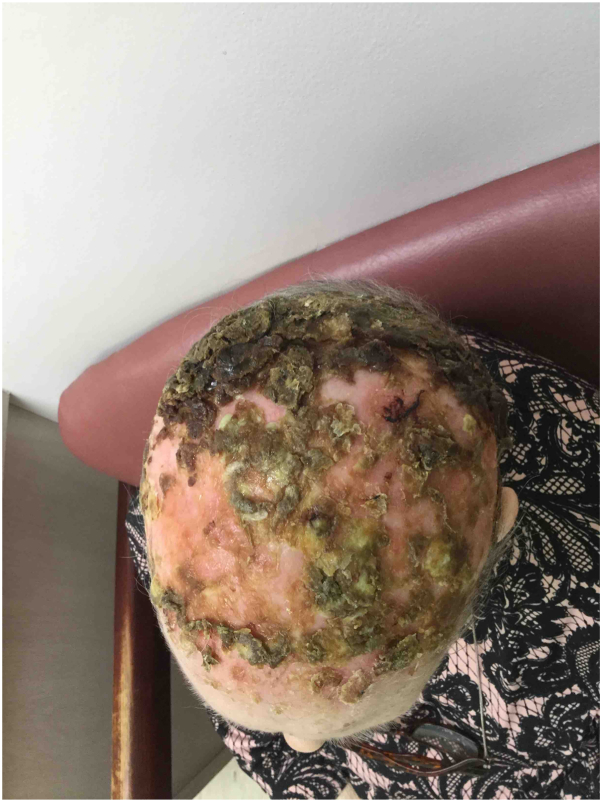
Erosive pustular dermatosis of the scalp—superior view severe crusting with erythema and alopecia of the scalp, affecting the frontal, temporal, and occipital regions.

**Fig 2:**
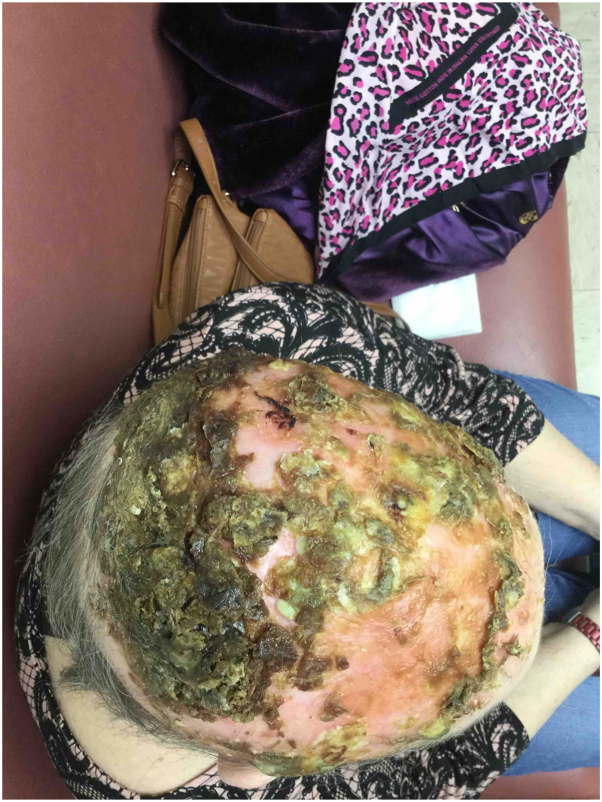
Erosive pustular dermatosis of the scalp—lateral view severe crusting with erythema and alopecia of the scalp, affecting the frontal, temporal, and occipital regions.

An 81-year-old female with a medical history of diabetes, iron deficiency anemia, heart arrhythmias treated with a pacemaker, hypertension leading to stage 4 kidney disease, basal cell carcinoma, squamous cell carcinoma, and plaque psoriasis without involvement of the scalp, presented to the dermatology clinic in 2017. She reported severe crusting with erythema and alopecia of the scalp, affecting the hairline on the forehead, around the ears, and the temporal, frontal, and occipital regions (Figs 1 and 2). On physical examination, the affected areas were covered with large crusts, beneath which erythema and thick pus were observed upon squeezing.

The patient noted that these scalp findings began rapidly in 2008, plateaued, and then remained in this condition unchanged for 9 years. Previous treatments with oral minocycline and topical dapsone were ineffective, while topical bacitracin provided some relief by softening the crusts. Biopsy performed at an outside hospital showed chronic mixed inflammation with intraepidermal collections of neutrophils and direct immunofluorescence showed no evidence of a specific dermatosis. Based on these findings and the clinical presentation, a diagnosis of EPDS was made.

**Fig 3:**
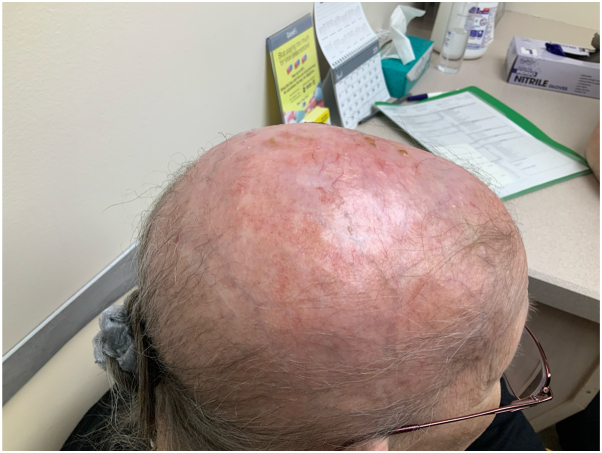
Resolution of erosive pustular dermatosis of the scalp—lateral view near-complete resolution of erosive pustular dermatosis of the scalp 6 months after initiating dialysis, showing significant improvement in the temporal and occipital regions.

**Fig 4:**
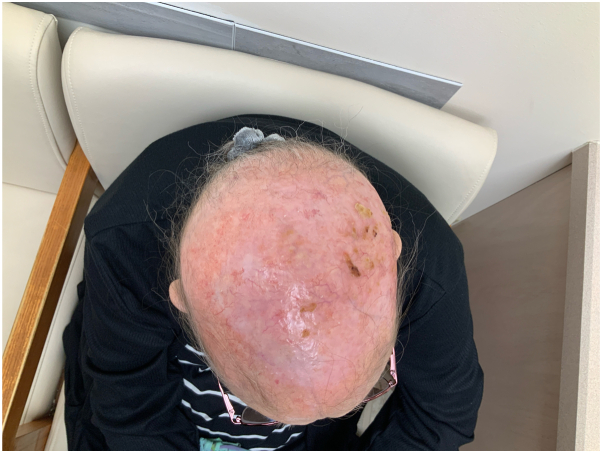
Resolution of erosive p dermatosis of the scalp—superior view near-complete resolution of erosive pustular dermatosis of the scalp 6 months after initiating dialysis, showing significant improvement in the frontal region.

Following the diagnosis in 2017, the patient was treated with tacrolimus ointment, clindamycin gel, and topical steroids. However, she discontinued topical steroids due to scalp atrophy, and these treatments offered minimal symptom relief. As her kidney disease progressed to stage IV, the patient began hemodialysis 3 days a week in September 2023. Remarkably, she experienced significant improvement shortly after starting hemodialysis. At a follow-up visit in March 2024, physical examination demonstrated near-complete resolution of her EPDS as seen in (Figs 3 and 4). Lab evaluation following inhiation of hemodialysis consistently revealed an elevated blood urea nitrogen/creatine and her iron deficiency anemia continued to be corrected with iron supplementation. There were no other electrolyte abnormalities or vitamin deficiencies recorded. It is important to note that health care providers were also assisting the patient with her scalp wound care weekly.

## Discussion

Traditionally, treatment of EPDS has involved the use of topical and systemic steroids. However, there is growing evidence supporting the efficacy of alternative treatments that may offer comparable benefits with potentially fewer adverse effects and lower recurrence rates. Topical tacrolimus (0.1%) has emerged as a highly effective therapy for both initial treatment and maintenance, demonstrating not only resolution of lesions but also the recovery of skin atrophy and hair regrowth.^2^^,^^3^ Similarly, photodynamic therapy, including both 5-aminolevulinic acid and methyl 5-aminolevulinic acid, has shown promise in treating EPDS, with recurrences being manageable through additional photodynamic therapy sessions.^4^

In addition to these treatments, other modalities such as acitretin, isotretinoin, silicone gels, and topical dapsone have also proven effective with a low risk of adverse effects.^1^ Oral dapsone therapy is recommended for cases where EPDS is disseminated beyond the scalp, indicating its potential utility in more extensive disease presentations.^5^ Cyclosporine, although effective in a documented case, presents significant risks including hypertension and increased creatinine levels, making it a less desirable option, especially for elderly patients.^6^

In this case, conventional treatments provided minimal relief. However, following the initiation of dialysis for stage IV kidney disease, the patient experienced a notable improvement in her EPDS symptoms. This observation suggests a potential link between dialysis and the resolution of EPDS, warranting further investigation into the pathophysiological mechanisms involved. The resolution of EPDS with dialysis might be explained through several potential mechanisms. Dialysis can modulate the immune system by removing inflammatory mediators, thereby reducing scalp inflammation. This is particularly relevant given the chronic inflammatory nature of EPDS.^7^ Additionally, dialysis aids in the detoxification process by removing metabolic waste products that might exacerbate inflammatory skin conditions like EPDS. Improved hydration and nutritional status achieved through regular dialysis sessions can further enhance overall skin health and promote healing, addressing the systemic contributors to skin disease.^7^ These combined effects may contribute to the unexpected resolution of EPDS in patients undergoing dialysis. This case also highlights the need for exploring alternative treatment modalities for EPDS, especially in patients with contraindications to traditional therapies. The rarity of this occurrence in the literature highlights a gap in the current understanding and management of EPDS, encouraging clinicians to consider a broad spectrum of treatment options and to contribute further to growing literature on EPDS management.

## Conflicts of interest

None disclosed.
